# Lipofibromatous hamartoma of the median nerve

**DOI:** 10.1186/1749-799X-5-71

**Published:** 2010-09-28

**Authors:** Talal Al-Jabri, Sunil Garg, Ganapathyraman V Mani

**Affiliations:** 1 Department of Plastic Surgery, East and North Hertfordshire NHS Trust, AL7 4HQ, UK; 2 Upper Limb Unit, Southampton University Hospitals NHS Trust, Tremona Road, Southampton, Hampshire, SO16 6YD, UK; 3 Queen Mary's Hospital Sidcup, South London Healthcare Trust, DA14 6LT, UK

## Abstract

Lipofibromatous hamartoma is a rare tumour of peripheral nerves which is characterised by an excessive infiltration of the epineurium and perineurium by fibroadipose tissue. To the best of our knowledge, only approximately 88 cases are reported in the literature. We report a rare case of lipofibromatous hamartoma of the median nerve causing secondary carpal tunnel syndrome in a 25 year old patient. This patient was treated conservatively with decompression and biopsy and experienced a complete resolution of symptoms post-operatively. Magnetic resonance imaging may be used to diagnose this lesion as it has very distinctive characteristics. Multiple conditions have been associated with this lesion and a greater understanding of these associations may clarify the pathogenesis. The architecture of the tumour makes excision very challenging and the surgical management remains controversial. A review of the literature regarding the etiology, pathogenesis and surgical management of lipofibromatous hamartoma is included.

## Introduction

Lipofibromatous hamartoma (LFH) was first described by Mason (1953) as a rare and slow-growing, benign neoplasm involving the peripheral nerves and their branches [1]. In LFH there is excessive proliferation of fibroadipose tissue which infiltrates the epineural and perineural elements of peripheral nerves thereby surrounding and separating nerve fascicles. This gives rise to a pathognomonic serpiginous, 'cable-like' appearance on magnetic resonance imaging (MRI) [2]. Most often LFH develops in the median nerve with a predilection for the carpal tunnel [3,4] however there are reports of LFH involving the ulnar, radial, sciatic and plantar nerves [3,5-7]. LFH is considered to be congenital in origin and has been commonly associated with macrodactyly and other conditions at birth. The surgical management remains controversial with some authors recommending decompression of the surrounding tissues, decompression and debulking of the fibrofatty sheath, microsurgical dissection and excision with or without cable grafts [8-11]. We report a rare case of LFH causing secondary carpal tunnel syndrome (CTS) in a 25-year-old gentleman which was treated successfully with decompression.

## Case Presentation

A 25-year-old Caucasian gentleman presented to our clinic with a 12 month history of worsening dysaesthesia and paraesthesia affecting the thumb and index finger of his right dominant hand. There were no deficits in motor function affecting the right hand. The patient reported no medical antecedents, apart from an apparent ganglion of his right wrist.

Physical examination revealed a soft, non-tender, subcutaneous 2 × 1 cm mass over the volar aspect of the right wrist with no atrophy of the thenar eminence. Altered sensation in the distribution of the median nerve of the right hand was detected by static two point discrimination and the use of the Semmes-Weinstein monofilament test. Tinel's sign and Phalen's test were both positive. Somatic examination did not reveal any signs of neurofibromatosis or lymph node tumefaction.

**Figure 1:**
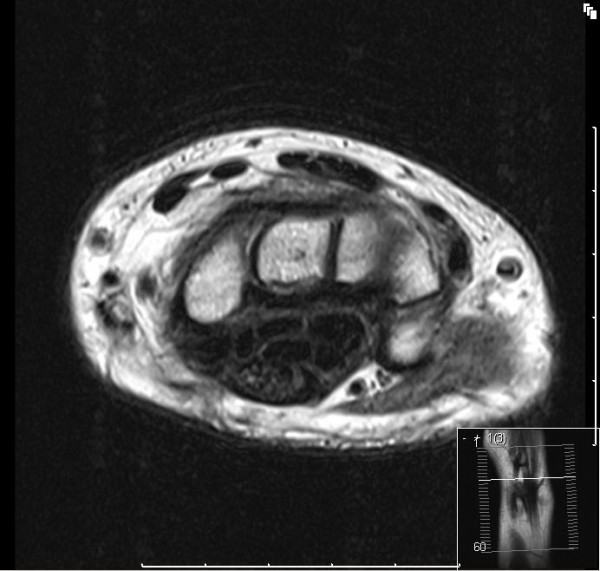
**MRI scan showing the typical 'cable-like appearance' on T1 axial section at the base of metacarpals**.

**Figure 2:**
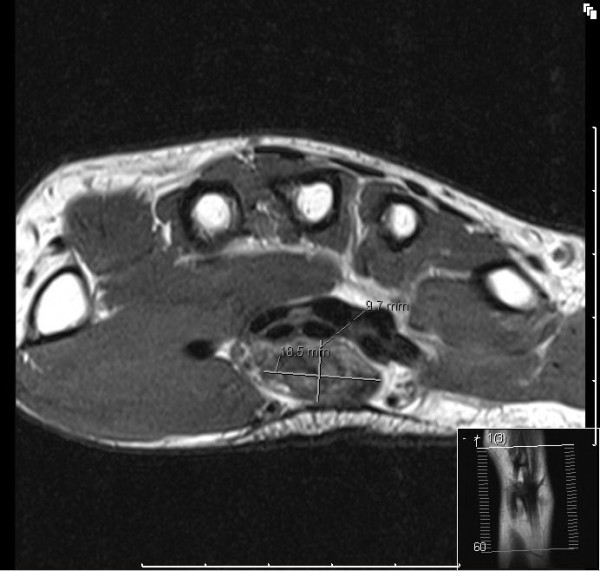
**MRI scan showing the suspicious swelling (18.5 mm × 9.7 mm) within the carpal tunnel**.

Nerve conduction studies inferred a reduction in median nerve conduction at the wrist. Radiographs of the hand and wrist did not show any ectopic calcifications or bony abnormalities. The MRI scan revealed an 18.5 × 9.7 mm volar mass within the carpal tunnel which was initially thought to be a ganglion cyst compressing the median nerve (Figures 1 and 2). On the basis of this surgical exploration and decompression was recommended.

**Figure 3:**
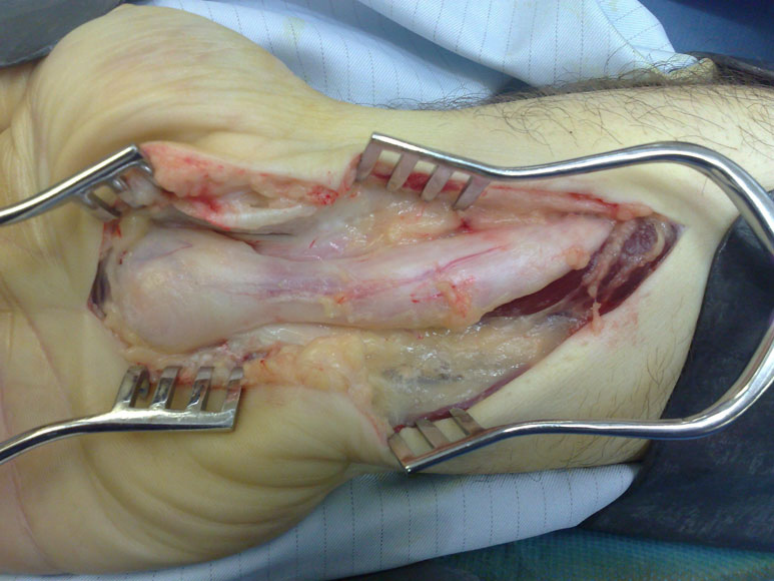
**Intra-operative view of the enlarged right median nerve with fibroadipose tissue proliferation**.

Under general anaesthetic, a longitudinal incision was made at the wrist. After dividing the transverse carpal ligament a grossly enlarged median nerve was observed. To facilitate exploration, the incision was extended up to the middle of the forearm and distally into the palm. The nerve was yellow and pink with longitudinal fatty streaks within the nerve fibres. These fatty streaks extended from the distal third of the forearm to the distal edge of the carpal tunnel (Figure 3). Excision of the adipose tissue between the nerve fascicles was impossible due to the risk of causing neuronal injury. A biopsy of the nerve was taken and the carpal tunnel was successfully decompressed.

Histological examination revealed non-neoplastic fibrofatty elements infiltrating the perineurium with mature fibrous and adipose tissue separating nerve fascicles. There was no neural hypertrophy. A histological diagnosis of LFH was made. The postoperative period was unremarkable and the patient experienced a complete resolution of his symptoms. At the 6 month follow-up appointment he remained asymptomatic.

## Discussion

LFH is a rare, benign overgrowth of fibroadipose tissue within a nerve sheath. It is most common amongst male Caucasians though there is a slight predominance amongst females when macrodactyly is present [11]. Clinical presentation is often with a progressive palmar tumefaction and a median compression neuropathy during childhood [4]. The differential diagnosis includes traumatic neuromas, ganglion cysts, lipomas and vascular malformations.

LFH can be diagnosed on MRI and this may negate the need for a biopsy [12,13]. On the axial image the nerve looks enlarged and nerve fascicles can be identified distinctly interspersed within fibroadipose tissue. This has been described as a 'cable-like' appearance and is pathognomonic for LFH (Figures 1 and 2). Our case has the typical 'cable-like' appearance however it was a retrospective finding. Although MRI is crucial for diagnosis and surgical planning, a recent Swedish study showed that MRI could only localise and diagnose a nerve tumour in the upper extremity in 75% of cases [2]. This emphasises the often overlooked limits of modern imaging and the need for good clinical judgement in surgical planning.

The etiology and pathogenesis of this lesion remains obscure and the use of numerous pseudonyms in the literature attest to this confusion (e.g. 'intraneural lipoma' [14], 'lipofibroma' [15] and 'fatty infiltration of the median nerve' [16]). Indeed, Johnson and Bonfiglio first introduced the term LFH and it is now considered the most appropriate [10].

LFH most commonly involves the median nerve though it is unclear as to why this predilection exists [10,17]. It has been suggested that chronic microtrauma to the median nerve from the carpal ligament or pressure from an abnormally developing flexor retinaculum can initiate a reactive process which culminates in the development of this tumour. This theoretical mechanism shares a common notion with the pathogenesis of a traumatic neuroma however, it is important to note that LFH usually extends beyond the carpal ligament [18]. Some clinicians are of the opinion that if this were the sole mechanism responsible for the development of LFH then one would expect LFH to develop in other confines within the body such as the lateral femoral cutaneous nerve under the inguinal ligament however, this is not the case [18]. Silverman and Enzinger examined the clinicopathological characteristics of 26 cases of LFH and found 25 of the 26 cases to be distributed in the median nerve and hand with only 1 case involving the foot [3].

LFH is often considered to be congenital in origin as most cases manifest in children and generally follow an indolent course [3,17,18]. However, some authors have demonstrated that a minority of patients will not present until at least the third decade [4]. Patil recently reviewed the literature and found that only approximately 88 cases of LFH have been reported and 33 of these were associated with macrodactyly [19]. This association is well addressed in the literature and has prompted speculation that LFH may be associated with neurofibromatosis, though none of the cases reported have a family history of neurofibromatosis [3]. It is also interesting to note that several cases of LFH have been associated with exostoses and ectopic calcifications [20]. Gigantism in patients with neurofibromatosis is associated with the development of osteochondromas further seeding this speculation of a possible association. Nevertheless, histological examination of nerves from patients with neurofibromatosis does not show the fatty infiltration seen in LFH and patients with neurofibromatosis usually present with numerous hallmarks of the disease. It has been suggested that a dysgenetic disorder similar to neurofibromatosis may ultimately be responsible for LFH [18,19] however further research is required to substantiate this. Silverman and Enzinger have also postulated that there may be a genetic difference between LFH associated with and without macrodactyly [3]. Finally, lipomas and vascular tumours have also been associated with LFH. Indeed, Al-Qattan suggested that an unidentified trophic factor which may cause LFH may also be responsible for the associated conditions [21]. A greater understanding of all these associations may be key to revealing the pathogenesis of LFH [21].

Histologically, LFH shows the presence of mature fibroadipose tissue intermingled with nerve fascicles thus expanding the epineurium. An onion bulb-like appearance is seen due to increased perineurial cells and perineurial fibrosis [4]. The architecture of the tumour makes complete excision very challenging and the surgical management remains controversial.

Radical excision of the mass with fascicular cable graft repair generally yields poor results [9]. Microsurgical dissection has previously been unsuccessful [22] although recently Clavijo-Alvarez, et al. were able to show preserved neurological function following intraneural fascicular dissection and nerve grafting [11]. A more conservative approach with decompression and debulking of the fibrofatty sheath yields more positive results [8,10]. Warhold, et al. reported an improvement in symptoms in patients treated with carpal tunnel decompression [4,8,10]. Severe sensory deficits with attempted surgical exicisions have been reported [4,9] although, there are rare reports of radical excisions producing no neurological deficits in children. Postoperative analysis of these patients showed the presence of Martin-Gruber anastomosis (motor nerve crossover from the median to the ulnar nerve in the distal forearm) [9]. Therefore, in severe cases and where a Martin-Gruber anastomosis is demonstrated, a more extensive excision may be possible without causing significant neurological deficit. Our patient had complete resolution of symptoms following decompression and nerve biopsy.

In conclusion, LFH is a rare tumour with a pathognomonic appearance on MRI. A diagnosis of LFH should always be considered in patients with palmar tumefaction and a median compression neuropathy. Sound clinical judgement is required in surgically planning the treatment of these patients. There is no definitive treatment for LFH however a conservative approach is commonly advocated with successful results from decompression as in our patient.

## Abbreviations

CTS: Carpal Tunnel Syndrome; LFH: Lipofibromatous Hamartoma; MRI: Magnetic Resonance Imaging.

## Consent

Written informed consent was obtained from the patient for the publication of this article and the accompanying images. A copy of the written consent is available for review by the Editor-in-chief of this journal.

## Competing interests

The authors declare that they have no competing interests.

## Authors' contributions

TAJ, SG and GVM made substantial contributions to the acquisition of data and its analysis. TAJ wrote the manuscript. SG and TAJ edited the manuscript. TAJ, SG and GVM were involved in the surgical management of the patient. All authors have approved the publication of this case report.
